# Students’ Class Perceptions and Ratings of Instruction: Variability Across Undergraduate Mathematics Courses

**DOI:** 10.3389/fpsyg.2021.576282

**Published:** 2021-06-10

**Authors:** Jesse L. M. Wilkins, Brett D. Jones, Lee Rakes

**Affiliations:** ^1^School of Education, Virginia Tech, Blacksburg, VA, United States; ^2^Assessment and Institutional Research, Virginia Military Institute, Lexington, VA, United States

**Keywords:** class perceptions, mathematics education, multilevel modeling, MUSIC model of motivation, student evaluations of teaching

## Abstract

The primary purpose of this study was to examine whether students’ motivation-related perceptions of mathematics courses were related to their ratings of instruction while controlling for their academic major, type of math class, and expected grade in the class. We investigated these relationships at both the student- and class-level because little is known about whether students’ motivation-related perceptions vary across mathematics courses and whether this variance is related to overall class ratings of instruction. The sample included 795 students nested within 43 different mathematics course sections. Students provided their course perceptions of autonomy, utility value, expectancies for success, situational interest, instructor caring, expected grade, and their overall perceptions of the course and instructor. Multilevel modeling techniques were used to investigate potential student- and class-level effects as well as compositional effects. Students’ class perceptions varied significantly across mathematics courses. In addition, students’ motivation-related course perceptions were positively related to their instructor and course ratings at both the student-level and class-level; however, the strength of these relationships sometimes varied across courses for some of the motivation-related perceptions. These results suggest that the motivational climate (i.e., the psychological environment) can affect students’ instructor and course ratings. Moreover, these findings suggest that instructors have some control over their instructor and course ratings through the teaching strategies that they implement. For example, they may be able to increase their ratings by implementing teaching strategies that support students’ autonomy, goals, success, interests, and relationships.

## Introduction

Students’ perceptions in a mathematics class are important because these perceptions can affect their motivation and engagement in the class, and subsequently, their learning and achievement (Middleton and Jansen, 2011; Christenson et al., 2012; Middleton et al., 2017). For example, students’ perceptions about the usefulness of the content, their ability to succeed in the course, and the level of caring relationships they have with others in the class are but a few of the many perceptions that can affect students’ motivation and learning (Wentzel and Miele, 2016). Not surprisingly, these types of course perceptions are also related to students’ ratings of the instructor and course (Jones, 2010). Although scholars have questioned whether student ratings of instruction are accurate measures of course quality (Kulik, 2001; Theall and Franklin, 2001; Carpenter et al., 2020), student ratings remain important at many institutions because they are often used for personnel decisions, such as annual reviews, merit raises, and promotion decisions (Miller and Seldin, 2014). Therefore, these ratings have serious consequences for instructors (Linse, 2017; Stroebe, 2020).

Although researchers have documented relationships between students’ class perceptions and their ratings of instruction in several different academic disciplines, studies of this relationship in undergraduate math classes are lacking. The study of college students in mathematics courses is useful because (a) students’ beliefs, attitudes, and value for mathematics tend to decline across secondary school (Wilkins and Ma, 2003; Watt, 2004; Chouinard and Roy, 2008); and (b) students sometimes perceive mathematics courses to be difficult, tedious, and boring (Gersten et al., 2009). It is important that students are motivated and engaged in mathematics classes because they are often required in colleges and universities–even for non-mathematics majors–because of the importance and usefulness of mathematics in other subject areas. Having a better understanding of how students’ motivation-related perceptions vary across courses could provide instructors with insights that could lead them to use more effective motivation strategies that could then lead to increased student engagement and learning. For example, if students’ success beliefs about a course are more important for non-mathematics majors than majors, instructors of those courses could identify teaching strategies that focus more specifically on success perceptions.

Researchers have studied the relationships among various class perceptions in math classes at the student-level (e.g., Jansen, 2006, 2008; Middleton, 1995, 2013; Skaalvik et al., 2017; Grigg et al., 2018). However, less is known about the extent to which students’ class perceptions relate to their ratings of instruction in different math courses, such as courses for math majors versus non-majors, and introductory versus advanced math courses. Examining the variation in student perceptions across classes and the interaction between student- and class-level perceptions requires studies that consider these multilevel contexts, and that employ multilevel statistical methods to model and examine these relationships (Marsh et al., 2012; Sloane and Wilkins, 2017).

The goal of this study was to examine the relationships between students’ class perceptions and their instructor and course ratings while controlling for their academic major, type of math class [e.g., upper-level courses, courses for non-science, technology, engineering, and mathematics (STEM) students], and expected grade in the class. We assessed these relationships at both the student-level and class-level because little is known about how students’ course perceptions vary at the class-level in the discipline of mathematics. We also compared the relationships between students’ perceptions and ratings of instruction at the student-level versus the class-level because differences would indicate potential compositional effects associated with courses. This study is important because few, if any, large-scale studies have been conducted to investigate the relationship between students’ class perceptions and perceptions of instruction in mathematics (Middleton et al., 2017).

## Students’ Perceptions of Class

Within any particular class, students’ perceptions can vary, sometimes significantly (Wolters, 2004). Many factors can affect students’ class perceptions, including the instructional activities (Urdan and Schoenfelder, 2006) and the social culture of the classroom (Gresalfi et al., 2012; Spearman and Watt, 2013). In this section, we explain the perceptions we included in this study and describe the criteria we used to select these perceptions.

### Motivation-Related Class Perceptions

#### Perceptions Associated With the MUSIC Model of Motivation

**FIGURE 1:**
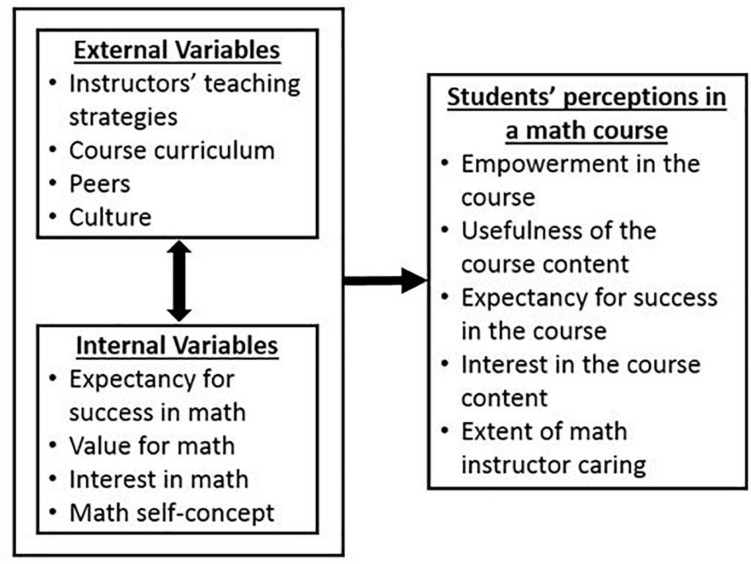
A few of the many possible antecedents of students’ perceptions in a mathematics course [Adapted from Jones (2018)].

In this study, we assessed students’ course perceptions associated with the five components of the MUSIC Model of Motivation (abbreviated as the MUSIC model, see Figure 1; Jones, 2009, 2018, 2020). The MUSIC model was developed specifically to provide instructors with evidence-based categories of instructional strategies that could be used to design instruction to motivate students to engage in learning. The five broad categories of instructional strategies correspond to five key principles of the MUSIC model: “The instructor needs to ensure that students:

(1) feel empowered by having the ability to make decisions about some aspects of their learning,
(2) understand why what they are learning is useful for their short- or long-term goals,
(3) believe that they can succeed if they put forth the effort required,
(4) are interested in the content and instructional activities, and
(5) believe that others in the learning environment, such as the instructor and other students, care about their learning and about them as a person” (Jones, 2018, p. 9).

MUSIC is an acronym based on the initial sounds of the titles of these categories: Empowerment, Usefulness, Success, Interest, and Caring.

We chose to examine students’ perceptions related to thefive MUSIC model components for several reasons. First, the MUSIC model is a multi-level theory (Jones, 2018, 2020) because it explains the effects of internal variables (e.g., beliefs, affect) on students’ MUSIC perceptions and how these relationships are affected by external variables (e.g., instructors, curriculum, peers, culture; see Figure 1). Therefore, the model provides a useful framework for examining the multilevel nature of motivation within and across different contexts, in particular, students nested within classrooms. Second, we wanted to examine students’ perceptions that can be changed by an instructor or specific interventions, which is true of the MUSIC model components (Reeve et al., 2004; Wang and Eccles, 2013; McGinley and Jones, 2014; Turner et al., 2014; Hulleman et al., 2017). Third, we wanted to include perceptions derived from multiple theoretical perspectives to increase the likelihood that we would capture the diversity of possible perspectives that may vary across courses. The MUSIC model meets this criterion because the model was developed based on teaching strategies extrapolated from theories that focus on students’ motivation in educational settings (Jones, 2009, 2016, 2018). Fourth, we wanted to select students’ perceptions that were measurable and the college student version of the MUSIC^®^ Model of Academic Motivation Inventory (Jones, 2012/2021, abbreviated as the MUSIC Inventory) has been shown to produce scores valid for use with many different populations including undergraduate students in the United States (Jones and Skaggs, 2016; Chittum et al., 2019; Jones, 2019), in China (Jones et al., 2017), and Colombia (Jones et al., 2017). Finally, we wanted to limit the number of perceptions included in our study to avoid assessing perceptions of similar constructs (e.g., self-efficacy and expectancy for success); yet, we wanted to include enough constructs to capture the diversity of possible perspectives that may exist. The MUSIC model was developed as a multidimensional and parsimonious model and the results of factor analyses have confirmed that the five MUSIC model components are correlated, yet distinct, in samples of college students (Jones and Wilkins, 2013; Jones and Skaggs, 2016; Jones et al., 2017), students in professional schools (e.g., pharmacy students, Pace et al., 2016; medical students, Gladman et al., 2020; veterinary medical students, Jones et al., 2019), middle and high school students (Parkes et al., 2015; Schram and Jones, 2016; Chittum and Jones, 2017), and elementary school students (Jones and Sigmon, 2016).

#### The Five Perceptions Related to the MUSIC Model

Students perceive a class to be *empowering* when they have freedom and control over some aspects of their learning environment. Teaching strategies related to the empowerment component (e.g., providing students with choices) are often derived from self-determination theory (Deci and Ryan, 2000), in which autonomy is conceptualized as a basic human psychological need. When their need for autonomy is met, students are more likely to perform at higher levels than when their autonomy need is not met (Cerasoli et al., 2016). It is important to note that although empowering students can meet their need for autonomy, it is not conceptually equivalent to *autonomy support*, which is a more inclusive construct than empowerment that includes constructs such as interest, intrinsic motivation, competence, relatedness, sense of challenge, and intrinsic goals (for a review, see Su and Reeve, 2011).

Students perceive a course to be useful when the content relates to their personal goals. The teaching strategies included in the *usefulness* component were derived from a variety of constructs and theories, such as the utility value component of the expectancy-value theory (Wigfield and Eccles, 2000), future-time perspective (Lewin, 1942; Nuttin and Lens, 1985), and goal setting theories (Locke and Latham, 2002). When students understand the usefulness of what they are learning, they are more likely to be motivated and perform at higher levels than when they do not (Patall et al., 2013; Hulleman et al., 2017).

The *success* component of the MUSIC model refers to strategies that help students perceive that they can succeed if they put forth the appropriate effort. Perceptions of success and competence are central to many theories of motivation, but a short list of theories includes self-efficacy (Bandura, 1997), self theories of intelligence (Dweck, 1999), theories that include expectancy constructs (e.g., Atkinson, 1964; Wigfield and Eccles, 2000), and self-worth theory (Covington, 1992). When students believe that they can succeed at an activity they are more likely to choose to engage in that activity, put forth effort in that activity, and persist at that activity (Bandura, 1997; Durik et al., 2015; Dietrich et al., 2017).

Students perceive a class to be *interesting* when it captures their attention and they are emotionally engaged in the content. Researchers have differentiated between situational interest (which is context dependent) and individual interest, which is developed over time as students acquire more knowledge and greater value for the activity or topic (Hidi and Renninger, 2006). Interest and emotions have been studied through a variety of theories and constructs, including the four-phase model of interest development (Hidi and Renninger, 2006), arousal (Duffy, 1957), flow (Csikszentmihalyi, 1990), and intrinsic motivation (Deci, 1975). When students are more interested in the course content, they tend to be more motivated as evidenced by increased attention, pursuing goals conducive to learning, and selecting related courses in the future (Schraw and Lehman, 2001; Ainley et al., 2002; Harackiewicz et al., 2008). In particular, students’ interest in mathematics has been found to be one of the strongest predictors of students’ quality of experience in mathematics class (Schiefele and Csikszentmihalyi, 1995).

The *caring* component of the MUSIC model refers to the quality of relationships that students have with the instructor and other students in the class. Students perceive others as caring when the instructor (or others in the learning environment) care about whether they succeed in the coursework and about their well-being. Teaching strategies related to the caring component have resulted from researchers who study social relationships with constructs such as the need for relatedness (Deci and Ryan, 2000), belonging (Baumeister and Leary, 1995), attachment (Bowlby, 1969; Ainsworth, 1979), and caring (Noddings, 1984, 2002; Wentzel, 1999). Caring constructs have been shown to be related to a variety of motivation-related constructs, including self-efficacy, intrinsic motivation, task value, and engagement (Furrer and Skinner, 2003; Freeman et al., 2007; Quin, 2017).

### Instructor and Course Ratings

Undergraduate courses have been, and continue to be, evaluated using student ratings of instruction (also referred to as student evaluations of teaching) to some extent (Marsh, 1987; Carpenter et al., 2020). Linse (2017) argues that student ratings will continue to be used because they provide feedback to improve instruction, they are useful in making personnel decisions (e.g., promotion and tenure decisions), and faculty generally agree that students’ perceptions should not be ignored.

Student ratings of the instructor and course are related to students’ motivation-related course perceptions. For example, Griffin (2016) found that students’ evaluations of teaching were significantly correlated with their perceptions of intrinsic motivation (correlations ranged from 0.31 to 0.64) and autonomy support (correlations ranged from 0.23 to 0.42) in college courses. In addition, Jones (2010) found that all five of the MUSIC model constructs were significantly correlated with instructor ratings (correlations ranged from 0.22 to 0.67) and course ratings (correlations ranged from 0.24 to 0.64) in an online and face-to-face course, with only a few exceptions for some subgroups. It is not surprising that student ratings of instruction are related to their motivational course perceptions because, as noted in the prior section, students’ perceptions of the MUSIC variables are related to a variety of motivational and achievement outcomes that can affect their perceptions of the instructor and course. It is not completely clear why students who are more motivated and engaged in a course rate the instructor and course higher; nonetheless, these variables are consistently correlated in many studies (Filak and Sheldon, 2003; Jones and Skaggs, 2016; Jones, 2019).

## Research Questions

The purpose of this study was to investigate the relationship between students’ class perceptions and their instructor and course ratings while controlling for their academic major, type of math class, and expected grade in the class. Prior studies have demonstrated relationships between these variables at the student level (e.g., Jones, 2010, 2019; Griffin, 2016; Jones and Skaggs, 2016), but studies are lacking that examine these relationships at the course level and that investigate the compositional effects to assess the differences between student- and class-level effects. Understanding these relationships is important in math courses because they serve as gateway courses for both math majors and many non-math majors. Our study addresses the following research questions.

(1) To what extent do students’ MUSIC perceptions vary across mathematics classes?
(2) At the student level, to what extent do students’ MUSIC perceptions of a mathematics course relate to their instructor and course ratings?
(3) At the class level, to what extent do students’ mean MUSIC perceptions of a mathematics course relate to their mean instructor and course ratings?
(4) Are there compositional effects (i.e., differences between student- and class-level effects) associated with students’ instructor and course ratings?

We controlled for academic major and type of math class because studies in other STEM disciplines have documented differences in students’ motivation-related perceptions between majors and non-majors (e.g., Shell and Soh, 2013). We also controlled for expected grade in the class because most studies have found that instructor ratings are correlated with students’ course grades (Beran and Violato, 2005; Greenwald and Gillmore, 1997; Marsh and Roche, 2000).

## Method

### Participants

All students involved in the study were enrolled in a mathematics course during a fall semester in a medium-sized college in the southeastern United States. Of the 47 mathematics courses offered at the institution that semester, students in 44 of the courses completed a paper survey. The complete data collected included 796 undergraduate students. One course containing only one student was removed from the data, which resulted in a working sample of 795 students nested within 43 different course sections. For some courses, there were multiple sections. For nine courses, the same instructor taught two sections; and in one case, the same instructor taught three sections of the same course; otherwise, course sections were taught by different instructors. Research methods and procedures for this study were conducted in accordance with human subjects guidelines and approved by our institution’s Institutional Review Board for research involving human subjects.

Of the 795 students, 9.9% were female, 85.4% were male, and 4.7% did not indicate their sex. These percentages were consistent with the overall percentages of males and females enrolled at the college during that semester (10.8% female, 89.2% male). The sample included individuals who identified as White (73.08%), Black or African American (6.04%), Hispanic (3.65%), Asian or Pacific Islander (7.55%), American Indian (0.50%), and Mixed or Other (4.03%), with 5.16% who did not provide their race/ethnicity. The students were 18.36% first year, 23.90% second year, 22.39% third year, and 29.81% fourth year, with 5.53% who did not report their class standing.

### Measures

Participants completed a paper survey during class that included items related to their MUSIC perceptions of the course, as well as other course-related information. After completing the survey, students completed a second survey with items related to their demographic information. This administration ordering strategy was used to help control for the effects of stereotype threat related to students’ gender and ethnicity (Steele, 1997).

#### Student-Level Variables

##### Motivation-related perceptions

We used the College Student version of the MUSIC^®^ Model of Academic Motivation Inventory (Jones, 2012/2021), which includes 26 items that measure students’ course perceptions related to the five motivation-related constructs listed in Table 1. The empowerment and usefulness scales have five items each, the success scale has four items, and the interest and caring scales have six items each. The inventory items were rated on the following scale: *1* = *Strongly disagree*, *2* = *Disagree*, *3* = *Somewhat disagree*, *4* = *Somewhat agree*, *5* = *Agree*, and *6* = *Strongly agree*. An example item from each scale follows: “I had control over how I learned the course content” (empowerment/autonomy), “In general, the coursework was useful to me” (usefulness/utility value), “I was confident that I could succeed in the coursework” (success/expectancy for success), “The coursework was interesting to me” (interest/situational interest), and “The instructor cared about how well I did in this course” (caring). Researchers have reported excellent Cronbach’s alpha values for each scale [the first values are from Jones and Skaggs (2016) and the second and third values are from Jones et al. (2017)]: 0.91, 0.82, 0.88 for empowerment; 0.96, 0.89, 0.93 for usefulness; 0.93, 0.87, 0.91 for success; 0.95, 0.93, 0.95 for interest; and 0.93, 0.88, 0.92 for caring. Researchers have also provided evidence of convergent, divergent, and predictive validity in samples of college students, including evidence that students’ MUSIC perceptions were significantly related to their effort and ratings of courses and instructors (Jones and Skaggs, 2016) and to cognitive and behavioral engagement (Jones et al., 2017).

**TABLE 1:** Constructs assessed by the MUSIC inventory.

MUSIC model constructs	Definitions The degree to which a student perceives that:	Related constructs
Empowerment	He or she has control of his or her learning environment in the course	Autonomy (Deci and Ryan, 1991)
Usefulness	The coursework is useful to his or her future	Utility value (Wigfield and Eccles, 2000)
Success	He or she can succeed at the coursework	Expectancy for success (Wigfield and Eccles, 2000)
Interest	The instructional methods and coursework are interesting	Situational interest (Hidi and Renninger, 2006)
Caring	The instructor cares about whether the student succeeds in the coursework and cares about the student’s well-being	Caring (Noddings, 1992)

*From “User Guide for Assessing the Components of the MUSIC^®^ Model of Academic Motivation,” by Jones (2012/2021), p. 5. Copyright 2012/2021 by Brett D. Jones. Reprinted with Permission.*

##### Students’ instructor and course ratings

Students provided a rating of the course instructor based on the following item-stem and scale: *My overall rating of the instructor for this course* (*1* = *Terrible*, *2* = *Very poor*, *3* = *Poor*, *4* = *Good*, *5* = *Very good*, *6* = *Excellent*). Similarly, students were asked to provide a rating of the course based on the following item-stem and scale: *My overall rating of the course* (*1* = *Terrible*, *2* = *Very poor*, *3* = *Poor*, *4* = *Good*, *5* = *Very good*, *6* = *Excellent*). These items have been used in other studies (e.g., Jones, 2010) and are similar to the items included on the mandatory course evaluation forms used at some universities. Scores from these items were used to create the variables INSTRUCTOR and COURSE, respectively. In this study, we used these ordinal measures as approximations of an underlying continuous variable. Research suggests that Likert scales with five or more categories can be analyzed as continuous with little effect to the robustness of the statistics (Johnson and Creech, 1983; Zumbo and Zimmerman, 1993; Norman, 2010; Sullivan and Artino, 2013). There were seven (0.9%) and five (0.6%) missing scores for instructor and course ratings, respectively. Scores were imputed using the mean score for the specific course section. This imputation technique was used to better preserve the course-level perceptions.

##### Expected grade

Students were asked about their expected grade in the course using the following item-stem and scale: *What is your grade in this course?* (*1* = *A*, *2* = *B*, *3* = *C*, *4* = *D*, *5* = *F*). Scores for the expected grade question were reverse coded so that a higher score represented a higher grade. These scores were used as a proxy for students’ expectations for success in the course, which is consistent with the focus of this study about students’ perceptions. Scores from this item were used to create the GRADE variable. Again, research suggests that ordinal measures with five or more categories can be analyzed as continuous with little effect on the results (Johnson and Creech, 1983; Zumbo and Zimmerman, 1993; Norman, 2010; Sullivan and Artino, 2013). There were 18 (2%) missing scores for this variable. In order to better preserve course-level perception, scores were imputed using the mean score for the specific course section.

##### Academic major

Students were asked to select their academic major from a list of the 14 primary majors at the institution. We collapsed these majors into seven major categories that combined majors with similar disciplinary content background and mathematics requirements: Mathematics (Applied Mathematics), Computer Science (Computer and Information Sciences), Engineering/Physics (Civil Engineering; Electrical and Computer Engineering; Mechanical Engineering; Physics), Life Science (Biology; Chemistry), Liberal Arts (English, Rhetoric and Humanistic Studies; History; Modern Languages and Cultures), Social Sciences (Economics and Business; International Studies and Political Science; Psychology), and Other (students for whom their reported major is missing or miscoded). We created seven dummy-coded variables for the seven major categories (1 = *major*; 0 = *non-major*).

#### Class-Level Variables

##### Mathematics course type

We created three dummy variables to categorize courses by general type: Advanced Mathematics, Non-STEM, and Core STEM. Courses categorized as Advanced Math were those upper-level courses primarily taken by mathematics majors, with a select few courses taken by engineering, computer science, or physics majors (these courses are offered at the 300 and 400 level). Courses categorized as Non-STEM are those courses primarily taken by non-STEM majors to meet the mathematics/quantitative requirement for graduation (these courses are offered at the 100 level). This category includes courses in pre-calculus, introductory probability and statistics, and introductory calculus for economics and business. Courses categorized as Core STEM are those courses that are core to most STEM majors. This category primarily consists of those courses in the calculus sequence, but also includes courses that are specific to STEM majors (e.g., *Matrix Algebra* for mathematics and physics majors, *Probability and Statistics for Engineers and Scientists* for engineering majors; these courses are offered at the 100 and 200 level). Three dummy-coded variables were created for the three course types (1 = *in category*; 0 = *not in category*).

##### Class mean for instructor, course, and grade

For each of the three student-level variables–INSTRUCTOR, COURSE, and GRADE–a class-level variable was created by calculating the mean for each course section, respectively, INSTRUCTOR MEAN, COURSE MEAN, and GRADE MEAN.

### Analysis

The analysis we used to assess the structure of the MUSIC model components is presented in the “Results” section. To investigate the potential effects associated with course differences related to students’ class MUSIC perceptions, we employed a two-level hierarchical linear model (HLM) (Raudenbush and Bryk, 2002). The use of an HLM allows for the simultaneous investigation of both within- and between-class relationships. For the two-level HLM, we first estimated an unconditional model with no student- or class-level predictors (i.e., a one-way random effects analysis of variance model) for each of the five MUSIC perceptions. This model enabled us to determine the existence and magnitude of the within- and between-class variance associated with each of the five perceptions of academic motivation (to answer Research Question 1). Next, at the student level, we estimated a within-class (Level 1) equation (see Eq. 1 in Appendix A) for each of the five perceptions of academic motivation that regressed each of the five perceptions on student-level predictors (to answer Research Question 2). Finally, at the class level, we estimated a between-class (Level 2) equation (see Eqs 2–6 in Appendix A) that regressed mean academic motivation on other class-level variables (to answer Research Question 3). See Appendix A for a detailed description of the equations and procedures we used for the two-level HLM.

**FIGURE 2:**
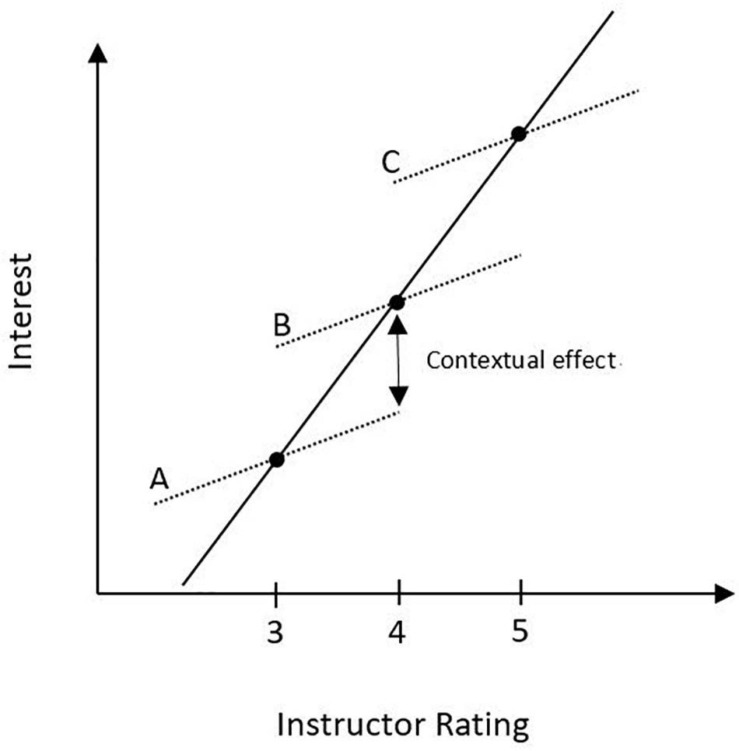
An illustration of a compositional effect associated with being a student in classroom B as opposed to a student in classroom A. In this case, the direction of the student-level effect and the class-level effect are in the same direction.

In addition to the student- and class-level effects, we examined the potential for compositional effects associated with being a member of different courses (to answer Research Question 4). Compositional effects represent the relationship associated with the aggregate of a student-level variable and the outcome variable after controlling for the student-level effect (see Raudenbush and Bryk, 2002). In our models, this was accomplished by including the student-level measures of INSTRUCTOR, COURSE, and GRADE in the Level 1 model as well as the aggregate or mean of these variables in the Level 2 model. The compositional effect was calculated as the difference in the class-level relationship (Level 2 effect) and the student-level relationship (Level 1 effect); and statistically, the magnitude of this difference reflects the strength of the compositional effect. Conceptually, it can be seen by comparing the average slope associated with the student-level effect or within-class relationships (represented by the dotted lines in Figure 2) with the slope of the line associated with the between-class relationship (see solid line in Figure 2). See Appendix B for further discussion of Figure 2.

## Results

### Factor Scores From the MUSIC Inventory

Based on the MUSIC model theory (Jones, 2009, 2018, 2020) and research (Jones and Wilkins, 2013; Jones and Skaggs, 2016; Pace et al., 2016), we hypothesized the MUSIC model as a correlated five-factor model and tested the validity of this model for the specific sample of students in this study using a Confirmatory Factor Analysis (CFA). Estimation of the model found the following fit indexes:χ^2^ (289) = 2050.60, CFI = 0.90, SRMR = 0.06, RMSEA = 0.09 (90% confidence interval [CI] = [0.085, 0.093]). Although, these indexes do not indicate a strong fit to the data, they do provide evidence of an adequate fit, and further corroborate findings from previous studies with undergraduate students (e.g., Jones and Wilkins, 2013; Jones and Skaggs, 2016; Jones et al., 2017). CFI values closer to 0.95 show good model fit (Hu and Bentler, 1999), but values greater than 0.90 usually show adequate model fit (Hoyle, 1995; Kline, 1998). SRMR values less than 0.05 are indicative of good model fit (Byrne, 2010), but SRMR values as high as 0.08 could represent good model fit (Hu and Bentler, 1999). Values for the RMSEA less than 0.05 represent good model fit, values between 0.05 and 0.08 represent reasonable model fit (Browne and Cudeck, 1993), and values between 0.08 and 0.10 represent “mediocre” fit (MacCallum et al., 1996, p. 134).

Because the cut-off values for fit indices should not be considered as the sole basis for accepting or rejecting a model (Marsh et al., 2004), we conducted further investigation of the individual scales’ dimensionality to test whether or not the items in the theorized groups represented a single factor. We examined the dimensionality of each of the five factors separately by conducting a separate principal components analysis for each factor along with a Velicer’s minimum average partial (MAP) test and a parallel analysis (compared to the 95th percentile based on 1,000 replications) (see O’Conner, 2000). Results of the MAP test and the parallel analysis suggested unidimensionality for each of the five groups of items, that is, they each formed a single factor. Lastly, we found the reliability of the scale scores to have strong internal consistency (α ≥ 0.89; see Table 2; Henson, 2001).

**TABLE 2:** Cronbach’s α for the five components of the MUSIC model.

Factor	α
Empowerment	0.89
Usefulness	0.92
Success	0.92
Interest	0.93
Caring	0.91
	

The strong evidence for the unidimensionality of each scale, the strong internal consistency of the scales, and the reasonable findings from the CFA, provided evidence that the scales constituted reliable and valid measures for the five components of the MUSIC model. We then created composite scores for each of the five MUSIC components by calculating the mean score for the item ratings. Descriptive statistics and intercorrelations for the five MUSIC components as well as the other student-level variables are presented in Table 3. Descriptive statistics and intercorrelations for class-level variables are presented in Table 4.

**TABLE 3:** Descriptive statistics and intercorrelations for student-level variables.

	M	U	S	I	C	Inst.	Course	Grade	Math	CS	EP	LS	LA	SS	Other
M		0.62	0.62	0.77	0.45										
U	0.60		0.60	0.74	0.36										
S	0.65	0.62		0.71	0.50										
I	0.76	0.73	0.75		0.41										
C	0.63	0.51	0.60	0.64											
Instructor	0.59	0.44	0.57	0.68	0.70										
Course	0.55	0.53	0.55	0.63	0.49	0.61									
Grade	0.19	0.21	0.50	0.29	0.19	0.26	0.31								
Math	0.10	0.08	0.11	0.13	0.09	0.09	0.14	0.19							
CS	0.04	−0.03	0.09	0.02	0.03	0.00	0.02	0.05	−0.08						
EP	−0.02	0.13	0.00	0.04	0.04	0.00	−0.01	−0.06	−0.34	−0.18					
LS	0.01	−0.05	−0.04	−0.03	−0.01	0.01	−0.03	−0.04	−0.14	−0.08	−0.32				
LA	0.01	−0.10	−0.02	−0.01	0.03	0.00	−0.03	−0.12	−0.08	−0.04	−0.19	−0.08			
SS	−0.04	−0.11	−0.05	−0.07	−0.09	−0.06	−0.04	−0.01	−0.18	−0.10	−0.40	−0.17	−0.10		
Other	−0.09	−0.04	−0.10	−0.12	−0.10	−0.06	−0.06	−0.02	−0.09	−0.05	−0.21	−0.09	−0.05	−0.11	

*M*	4.55	4.54	4.75	4.35	5.10	4.88	4.46	3.66	0.13	0.04	0.43	0.12	0.04	0.18	0.05
*SD*	0.98	1.12	1.10	1.19	0.90	1.27	1.27	1.13	0.34	0.20	0.50	0.32	0.21	0.38	0.22

*Correlations in the lower diagonal calculated at the student-level. Correlations in the upper diagonal calculated while accounting for class-level clustering effects. M = Empowerment, U = Usefulness, S = Success, I = Interest, C = Caring, Instructor = Instructor Rating, Course = Course Rating, Grade = Expected Grade, CS = computer Science, EP = Engineering/Physics, LS = Life Science, LA = Liberal Arts, SS = Social Science.*

**TABLE 4:** Descriptive statistics and intercorrelations for class-level variables.

	Instructor Mean	Course Mean	Grade Mean	Adv. Math	Core STEM	Non-major
Instructor Mean						
Course Mean	0.89					
Grade Mean	0.36	0.38				
Adv. Math	0.07	0.10	0.55			
Core STEM	0.06	0.14	−0.12	−0.48		
Non-major	−0.12	−0.24	−0.39	−0.46	−0.56	

*M*	4.86	4.46	3.71	0.28	0.37	0.35
*SD*	0.80	0.61	0.57	0.45	0.49	0.48

### Unconditional Model

Based on the unconditional model, the grand mean for the five measures of motivation were ${\hat{\gamma }}_{00}$ = 4.53 for Empowerment, ${\hat{\gamma }}_{00}$ = 4.51 for Usefulness, ${\hat{\gamma }}_{00}$ = 4.74 for Success, ${\hat{\gamma }}_{00}$ = 4.32 for Interest, and ${\hat{\gamma }}_{00}$ = 5.09 for Caring (see Table 5). On average, these measures represent a positive level of class perceptions for all five factors. To determine whether students’ MUSIC perceptions varied across classes, we examined the estimated between-course variance, ${\hat{\tau }}_{00}$, in each of the individual course means, β_0__*j*_, for each of these measures of motivational perception, and furthermore, whether this variance was significantly different from zero. Estimation of the unconditional models found statistically significant between-course variance for mean Empowerment (${\hat{\tau }}_{00}$ = 0.129), Usefulness (${\hat{\tau }}_{00}$ = 0.165), Success (${\hat{\tau }}_{00}$ = 0.196), Interest (${\hat{\tau }}_{00}$ = 0.313), and Caring (${\hat{\tau }}_{00}$ = 0.171) (see Table 5); thus, providing evidence that students’ MUSIC perceptions varied across courses.

**TABLE 5:** Unconditional models for the five components of the MUSIC model of motivation.

	Empowerment		Usefulness		Success		Interest		Caring	
					
Fixed effects	Effect	*SE*	Effect	*SE*	Effect	*SE*	Effect	*SE*	Effect	*SE*
Mean Class Motivation	4.529***	0.064	4.507***	0.072	4.744***	0.077	4.319***	0.093	5.087***	0.069
reliability estimate	0.721		0.712		0.757		0.819		0.809	

**Random effects**	**Variance**	***df***	**Variance**	***df***	**Variance**	***df***	**Variance**	***df***	**Variance**	***df***

Mean Class Motivation, ${\hat{\tau }}_{00}$	0.129***	42	0.165***	42	0.196***	42	0.313***	42	0.171***	42
Level 1, within class, ${\hat{\sigma }}^{2}$	0.833		1.113		1.040		1.121		0.655	
Attribution of variance										
Between class, ρ	0.134		0.129		0.159		0.218		0.207	
Within class, 1−ρ	0.866		0.871		0.841		0.782		0.793	

***p < 0.001.

We further examined the magnitude of the variation for each of the class perceptions by calculating a plausible value range for each of the grand means (Raudenbush and Bryk, 2002). For example, assuming normally distributed values of Empowerment for the 43 courses, then 95% of the course means would fall within the range ${\hat{\gamma }}_{00}$ ± 1.96(${\hat{\tau }}_{00}$)^1/2^, or 4.53 ± 1.96(0.129)^1/2^ = (3.83, 5.23); similarly, for Usefulness the range is (3.71, 5.31), for Success (3.87, 5.61), for Interest (3.22, 5.42), and for Caring (4.23, 5.90). Based on the metric for these variables, 1-to-6, a rating of 3.5 or higher would represent a more positive motivation-related perception; lower than 3.5 would represent a more negative average motivation. Based on these ranges, on average, student course perceptions are positive, however, the relative magnitude of positive ratings varies widely. For Interest, the range of plausible values dips below 3.5 indicating that for some courses the level of interest is not only relatively negative in comparison to other courses, but absolutely negative based on the 1-to-6 metric.

We can further ascertain the level of variance by considering the intraclass correlation coefficients (ICC) for each of the measures. The ICC represents the proportion of variance attributable to group membership, and often denoted as ρ where $\rho ={\hat{\tau }}_{00}\,/\,\left({\hat{\sigma }}^{2}+{\hat{\tau }}_{00}\right)$, and ${\hat{\sigma }}^{2}$ is the estimated pooled within-course variance. For Empowerment, ρ = 0.129/(0.129 + 0.833) = 0.134; or 13.4% of the variance in Empowerment is attributable to between-course differences. Similarly, the variance attributable to between-course differences for the other MUSIC components was 12.9% for Usefulness, 15.9% for Success, 21.8% for Interest, and 20.7% for Caring. These ICCs show that, on average, students’ perceptions differ across courses. Furthermore, the magnitude of the ICCs differs by factor, with Interest and Caring representing the highest ICCs, and Empowerment and Usefulness representing the lowest ICCs, with Success in the middle. It is important to note that although there is significant variability across courses in the motivation factors, the majority of variance is within-course or between-student, ranging from 78.2 to 87.1%. Thus, taken together, the amount of variance, plausible value ranges, and ICCs, provide evidence that, on average, students’ MUSIC perceptions differ by course.

### Conditional Models for the Five Components

Here we discuss the findings from estimating a two-level HLM for each of the five factors of the MUSIC Model.

#### Empowerment

Considering empowerment at the student level, on average, students majoring in mathematics had higher perceptions of empowerment than students majoring in either Engineering/Physics (β = −0.176, *p* < 0.05) or students in the Other category (β = −0.328, *p* < 0.10) (see Table 6). The students in the other majors (Computer Science, Life Science, Liberal Arts, and Social Sciences) were not found to have statistically different perceptions of empowerment than mathematics majors. Students’ grade expectation was not found to be related to their perceptions of empowerment.

**TABLE 6:** Final models for the five components of the MUSIC model of motivation.

	Empowerment		Usefulness		Success		Interest		Caring	
					
Fixed effects	Effect	*SE*	Effect	*SE*	Effect	*SE*	Effect	*SE*	Effect	*SE*
**Student-level effects**										
Mean Class Perception	4.527***	0.026	4.497***	0.611	4.748***	0.038	4.321***	0.029	5.086***	0.358
Instructor Rating	0.301***	0.038	0.217***	0.041	0.284***	0.040	0.388***	0.037	0.440***	0.032
Course Rating	0.215***	0.031	0.342***	0.039	0.215***	0.033	0.290***	0.038	0.065**	0.024
Expected Grade	0.008	0.028	0.101**	0.036	0.334***	0.038	0.105***	0.031	0.009	0.027
Major										
Computer Science	0.012	0.141	−0.494**	0.189	–0.013	0.166	–0.045	0.205	0.031	0.141
Engineer/Physics	−0.176*	0.085	−0.221***	0.063	−0.150^+^	0.086	−0.239***	0.066	−0.103^+^	0.056
Life Science	–0.108	0.127	−0.454***	0.130	−0.284*	0.124	−0.220^+^	0.124	–0.152	0.100
Liberal Arts	–0.120	0.160	−0.737***	0.165	–0.157	0.148	–0.052	0.149	–0.036	0.131
Social Sciences	–0.173	0.157	−0.465**	0.163	−0.348*	0.158	–0.116	0.162	−0.270*	0.116
Other	−0.328^+^	0.176	−0.324*	0.160	−0.492**	0.177	−0.532**	0.151	−0.299*	0.136
**Class-level effects**										
Advanced Math	−0.281***	0.064	−0.384**	0.115	−0.197^+^	0.104	−0.381***	0.070	–0.004	0.085
Non-STEM	–0.037	0.059	−0.383***	0.109	–0.008	0.087	−0.219**	0.067	–0.076	0.083
Instructor Mean	0.282**	0.079	0.112	0.137	0.204	0.127	0.524***	0.080	0.368**	0.110
Course Mean	0.279*	0.111	0.370^+^	0.216	0.263^+^	0.143	0.269*	0.126	0.102	0.125
Grade Mean	0.063	0.057	–0.163	0.099	0.363***	0.070	0.060	0.054	–0.071	0.057

**Random effects**	**Variance**	**df**	**Variance**	**df**	**Variance**	**df**	**Variance**	**df**	**Variance**	**df**

Mean Class Perception	0.007	36	0.063***	34	0.040***	34	0.013*	34	0.049***	36
Instructor Rating	0.020	41	0.025^+^	39	0.021**	39	0.015*	39	0.019***	41
Course Rating	*ns*	na	0.023**	39	0.012*	39	0.026***	39	*ns*	na
Grade	*ns*	na	0.016*	39	0.024***	39	0.006*	39	*ns*	na
Level 1, within class	0.540		0.700		0.524		0.568		0.341	
**Variance explained**										
Between class	0.946		0.618		0.796		0.958		0.713	
Within class	0.376		0.371		0.496		0.493		0.479	

^+^*p* < 0.10; *p < 0.05; **p < 0.01; ***p < 0.001.

**FIGURE 3:**
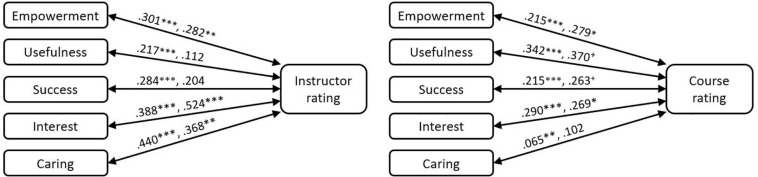
Relationships between students’ MUSIC perceptions and instructor and course rating. On each arrow, the first number represents the student-level effect and the second number represents the class-level effect. ^+^*p* < 0.10, ^∗^*p* < 0.05, ^∗∗^*p* < 0.01, ^∗∗∗^*p* < 0.001.

Controlling for student major and expected grade, on average, student instructor ratings (β = 0.301, *p* < 0.001) and course ratings (β = 0.215, *p* < 0.001) were both found to have a positive and statistically significant relationship with perceptions of empowerment (see Figure 3 and Table 6). The relationship between instructor ratings and empowerment were found to vary across courses. Calculating a 95% plausible value range, the differentiating effects of instructor ratings fall within the range of 0.160 to 0.442. So, although there is significant variability, in general, the relationship is consistently positive. The relationship between course ratings and empowerment was not found to vary by course. The student-level variables were found to predict 37.6% of the within-class variance in student perceptions of empowerment.

At the class level, we investigated the effects of the type of course (i.e., advanced mathematics, non-STEM, or core STEM). Overall, students in the core STEM courses were found to have a more positive perception of empowerment than the students in the advanced math courses (β = −0.281, *p* < 0.001). No difference was found between core STEM courses and Non-STEM courses. Mean Grade expectation was not found to be related to class-level empowerment. Mean instructor rating (β = 0.282, *p* < 0.01) and mean course rating (β = 0.279, *p* < 0.05) were found to have a positive and statistically significant relationship with class level empowerment (see Figure 3 and Table 6). Overall, class-level variables predicted 94.6% of the variance in between-course differences in perceptions of empowerment. The magnitude of the relationship at the class-level was not found to be different from that at the student level suggesting no compositional effects associated with course.

#### Usefulness

Student-level variables were found to predict 37.1% of the within-class variance in student perceptions of usefulness. At the student level, on average, non-mathematics majors had lower perceptions of usefulness for the mathematics course in which they were enrolled (see Table 6): Computer Science (β = −0.494, *p* < 0.01), Engineering/Physics (β = −0.221, *p* < 0.001), Life Science (β = −0.454, *p* < 0.001), Liberal Arts (β = −0.737, *p* < 0.001), Social Sciences (β = −0.465, *p* < 0.01), and Other (β = −0.324, *p* < 0.05). In addition, a positive and statistically significant relationship between student grade expectation and perceptions of usefulness was documented (β = 0.101, *p* < 0.01). This relationship was found to significantly vary by class (95% plausible value range: −0.147, 0.349).

Controlling for student major and expected grade, on average, a positive and statistically significant relationship was found between student instructor rating (β = 0.217, *p* < 0.001), course rating (β = 0.342, *p* < 0.001) and student perceptions of course usefulness (see Figure 3 and Table 6). These relationships were found to significantly vary by course. A 95% plausible value range for the differentiating effects of instructor ratings was expected to be in the range of −0.093 to 0.527; for course ratings, the range was 0.045 to 0.639. Evident from these ranges is the wide variability for these relationships, for example, in some classes we could expect the relationship with instructor ratings to be reversed as the range includes negative values and in other cases the relationship could be expected to be negligible as the range also includes zero.

At the class level, overall, students in the core-STEM courses were found to have more positive perceptions of the usefulness of the course than students in both advanced mathematics courses (β = −0.384, *p* < 0.01) and non-STEM mathematics courses (β = −0.383, *p* < 0.001). Mean expected grade was not found to be related to mean perceptions of course usefulness. Mean course rating had a positive and marginally significant relationship with class-level usefulness (β = 0.370, *p* < 0.10; see Figure 3 and Table 6). Mean instructor rating was not found to have a statistically significant relationship with mean class perceptions of usefulness. Class-level variables predicted 61.8% of the between-course variance in perceptions of usefulness. The magnitude of the relationships for course and instructor ratings at the class-level were not found to be different from those at the student level suggesting no compositional effects associated with class.

#### Success

Student-level variables were found to predict 49.6% of the within-class variance in student perceptions of success. At the student level, students majoring in engineering/physics (β = −0.150, *p* < 0.10), life sciences (β = −0.284, *p* < 0.05), social sciences (β = −0.348, *p* < 0.05), or other (β = −0.492, *p* < 0.01) were found to have lower perceptions of success than students majoring in mathematics. Interestingly, students with a liberal arts major were not found to have different perceptions of success from those majoring in mathematics (see Table 6). This was also true for computer science majors. A positive and statistically significant relationship between student grade expectation and perceptions of success was also documented (β = 0.334, *p* < 0.001). This relationship was found to significantly vary by class (95% plausible value range: 0.030, 0.638).

Controlling for major and expected grade, on average, student instructor rating (β = 0.284, *p* < 0.001) and course rating (β = 0.215, *p* < 0.001) were both found to have a positive and statistically significant relationship with student perceptions of success (see Figure 3 and Table 6). These relationships were also found to vary across courses. Plausible value ranges for the effects of instructor rating (0.000, 0.568) and course ratings (0.000, 0.430) both reflect wide variability in the relationships, but also suggest that in some classes we could expect practically no effect associated with ratings as the ranges include zero or values close to zero.

At the class level, students in advanced mathematics courses were found to have lower perceptions of success than students in the core-STEM courses (β = −0.197, *p* < 0.10). On average, mean grade expectation was found to have a positive and statistically significant relationship with average class-level perceptions of success (β = 0.363, *p* < 0.001). Mean course rating was found to have a positive and marginally significant relationship with class-level perceptions of success (β = 0.263, *p* < 0.10) and mean instructor rating was not found to be related to class-level success (see Figure 3 and Table 6). Class-level variables predicted 79.6% of the between-class variance in perceptions of success. No compositional effects were documented for perceptions of success.

#### Interest

Overall, student-level variables were found to predict 49.3% of the within-class variance in student perceptions of interest. At the student level, students majoring in engineering/physics (β = −0.239, *p* < 0.001) and life sciences (β = −0.220, *p* < 0.10) were found to have lower interest than students majoring in mathematics (see Table 6). Students categorized as Other were also found to have lower levels of interest than those majoring in mathematics (β = 0.104, *p* < 0.001). A positive and statistically significant relationship between student grade expectation and perceptions of interest was also documented (β = 0.334, *p* < 0.001). This relationship was found to significantly vary by class (95% plausible value range: −0.047, 0.257).

Controlling for major and expected grade, on average, a positive and statistically significant relationship was found between student instructor rating (β = 0.388, *p* < 0.001), course rating (β = 0.290, *p* < 0.001) and student perceptions of course interest (see Figure 3 and Table 6). These relationships were found to vary across classes. Plausible value ranges for the effects of instructor rating (0.148, 0.628) and course ratings (−0.026, 0.606) reflect the variability in the relationships, but for course ratings we could expect some classes where this relationship is inconsequential as some values in the range are close to zero. For instructor rating, it appears that in all classes that interest is positively related to instructor ratings, as the range does not include zero.

At the class level, students in advanced mathematics courses (β = −0.381, *p* < 0.001) and non-STEM courses (β = −0.219, *p* < 0.001) were found to have lower perceptions of interest than those students in core-STEM courses. Mean Grade expectation was not found to be related to class-level interest. Mean instructor rating (β = 0.524, *p* < 0.001) and mean course rating (β = 0.269, *p* < 0.05) were found to have a positive and statistically significant relationship with class-level interest (see Figure 3 and Table 6). Overall, class-level variables predicted 95.8% of the variance in between-course differences in perceptions of interest. Comparing the student-level effect (β = 0.388) and the class-level effect (β = 0.524) for instructor rating, we documented a marginally significant difference in these effects (*p* = 0.075). This difference suggests a compositional effect associated with course membership. In other words, on average, two students with instructor ratings differing by one unit, would differ in perceptions of interest by 0.388 of a unit. For comparison, consider two students with the same instructor rating, but in two different classrooms differing by one unit in *mean* instructor rating (e.g., see classrooms A and B in Figure 2); on average, the student in the course with the higher mean instructor rating would have an increase of 0.136 of a unit in perceptions of interest just by virtue of being a member of that course as opposed to the other course. The overall instructor perceptions of the class has a relationship with perceptions of interest beyond the individual student-level relationship.

#### Caring

Overall, student-level variables predicted 71.3% of the within-course variance in student perceptions of caring. At the student level, students majoring in Social Sciences were found to have lower perceptions of caring than students majoring in mathematics (β = −0.270, *p* < 0.05) (see Table 6). Students majoring in engineering/physics were also found to have lower perceptions of caring but the effect was only marginally significant (β = −0.103, *p* < 0.10). Students categorized as Other were also found to perceive lower levels of caring than those majoring in mathematics (β = −0.299, *p* < 0.05). Students’ expected grade was not found to be related to their perception of caring.

Controlling for major and expected grade, on average, students’ rating of instructor (β = 0.440, *p* < 0.001) and course (β = 0.065, *p* < 0.01) were found to have a positive and statistically significant relationship with students’ perception of caring (see Figure 3 and Table 6). Only the relationship with instructor ratings was found to vary across classes. The 95% plausible value range for this relationship (0.170, 0.710) suggests that although there is significant variability across courses we can expect a positive relationship between student perceptions of caring and the instructor ratings in all classes.

At the class level, there were no differences between students in advanced mathematics courses, core-STEM courses, and non-STEM courses; and mean expected grade did not have a significant relationship with caring. Mean instructor rating had a statistically significant relationship with class-level perceptions of caring (β = 0.368, *p* < 0.01; see Figure 3 and Table 6), but mean course rating did not. However, 47.9% of the between-course variance in perceptions of caring was predicted by class-level variables, primarily mean instructor rating. No compositional effects were documented for perceptions of caring.

### Associations Among the Five Factors of the MUSIC Model

In Table 3, we present the intercorrelations among the five MUSIC Inventory scales. The correlation coefficients in the lower diagonal were calculated at the student level, not taking into consideration the organizational unit (i.e., class section). The correlation coefficients in the upper diagonal were calculated using a two-level hierarchical multivariate linear model which takes into consideration the clustering effects associated with course. In general, the overall pattern for both sets of coefficients is consistent with expectations (Jones and Skaggs, 2016), as the five factors were moderately positively correlated. However, the associations between caring and the other four factors are all weaker when the clustering effects are controlled, with differences ranging from 0.10 for success to 0.23 for interest, whereas the differences are all within 0.04 for the coefficients for the other relationships. The coefficients in the upper diagonal provide a theoretically more valid representation of the relationships among the five factors as they reflect the multilevel structure and the clustering effects associated with the organizational unit. In addition, these findings are consistent with the large ICC associated with caring.

## Discussion

The purpose of this study was to examine whether students’ math class perceptions were related to their ratings of instruction while controlling for their academic major (at the student-level), type of math class (at the class-level), and expected grade in the class. We investigated these relationships at the student-level and class-level because little is known about how students’ MUSIC perceptions vary at the class-level in math courses. In this discussion, we address each of the four research questions in order.

### Variation Across Classes

Our first research question asked: To what extent do students’ MUSIC perceptions vary across mathematics classes? We found that students’ MUSIC perceptions–empowerment, usefulness, success, interest, and caring–varied significantly across the 43 undergraduate mathematics courses included in the study. We reached this conclusion by examining the amount of variance associated with the mean values, the plausible value ranges, and the ICCs. The extent to which students’ perceptions vary across courses (i.e., the amount of variance that is attributable to between-course differences) ranges from 12.9% for Usefulness to 21.8% for Interest. This variation in students’ perceptions across courses could be attributable to a variety of external factors associated with the course such as instructor quality, course curriculum, or content of course (see Figure 1). Because students’ MUSIC perceptions correspond directly to categories of MUSIC model strategies and given that students’ perceptions were found to vary by course, instructors can consider and choose instructional strategies to increase students’ motivation, engagement, and achievement outcomes. For example, to increase students’ perceptions of the usefulness of the course, instructors could select strategies related to the usefulness component of the MUSIC model (see Jones, 2009, 2018 for examples). In the following sections, we discuss the extent to which indicators of students’ instructor and course ratings are related to and predict student- and class-level variability in perceptions of motivation.

### Student-Level Relationships Between MUSIC Perceptions and Instructor and Course Ratings

Our second research question asked: At the student level, to what extent do students’ MUSIC perceptions of a mathematics course relate to their instructor and course ratings? Although we did document significant between-class variability in students’ class perceptions, recall that the majority of variance in perceptions is within-course or between-student, ranging from 78.2% for Interest to 87.1% for Usefulness. Here we discuss these between-student relationships. We found that, on average, students’ MUSIC perceptions were positively and significantly related to their instructor and course ratings (see Figure 3), a finding that is consistent with the results of other studies in undergraduate non-math courses (e.g., Jones, 2010, 2019; Griffin, 2016). This finding is important because our analysis controlled for students’ expected grade and major. Therefore, regardless of whether students expected to receive a high or low grade in the course, their MUSIC perceptions were related to their ratings of the instructor and course. This suggests that it may be possible for instructors who give lower grades to still obtain high instructor and course ratings if their students have higher MUSIC perceptions. Similarly, regardless of whether students were a math major or another major, their MUSIC perceptions were related to their instructor and course ratings. This finding is interesting because it indicates that instructors who teach non-math majors could still receive high instructor and course ratings if their students have higher MUSIC perceptions.

In sum, the overall implication is that if instructors use strategies consistent with the MUSIC model to increase students MUSIC perceptions, they may be more likely to receive higher instructor and course ratings. Because these results are correlational, we cannot assume that increasing students’ MUSIC perceptions will lead to increases in students’ instructor or course ratings. However, because these relationships are consistent with the MUSIC model theory (Jones, 2018, 2020), it is reasonable to speculate that if instructors increase the students’ MUSIC perceptions in the course, they will receive higher instructor and course ratings. Although experimental and quasi-experimental studies have demonstrated that it is possible to intentionally increase one or more of students’ MUSIC perceptions (Reeve et al., 2004; McGinley and Jones, 2014; Lin-Siegler et al., 2016; Hulleman et al., 2017), further research is needed to study the effects of these increases on students’ instructor and course ratings. Such experimental studies could provide evidence of a causal relationship between MUSIC perceptions and instructor and course ratings.

### Variation in Between-Student Relationships Across Classes

The strength of the between-student relationships between students’ MUSIC perceptions and their instructor rating varied across courses. Similarly, the strength of the between-student relationships associated with students’ MUSIC perceptions and their course rating varied across courses for usefulness, success, and interest. In other words, while overall, the relationships tended to be positive, these findings suggest that these relationships are not necessarily the same for all courses. Specifically, in some courses the relationship was positive and quite strong, while in others there was little to no relationship. Considering the relationships between students’ course ratings and their perceptions of empowerment and caring, it was found that these relationships did not significantly vary across courses; that is, there is a relatively constant positive relationship between course ratings and perceptions of empowerment, and course ratings and perceptions of caring. Although the relationship between course rating and empowerment and caring is consistent across courses, it does not imply that empowerment and caring are the most important factors related to course ratings. In fact, the relationship between course rating and the other MUSIC perceptions (i.e., usefulness, success, and interest) are higher in some courses than the relationship between course rating and empowerment and caring. In general, these results indicate that something about the courses and/or instructors–perhaps the content, the teaching methods, or the instructor’s characteristics–could potentially affect the relationships between students’ MUSIC perceptions and their instructor and course ratings (see Figure 1). In this study, our primary focus was to document the overall relationships between MUSIC perceptions and course and instructor ratings; therefore, we did not attempt to model the between-class differences in these relationships, but we highlight them to further emphasize potential differences in the interaction between variables across courses and as a potential focus for future research.

### Class-Level Relationships Between MUSIC Perceptions and Instructor and Course Ratings

Our third research question asked: At the class level, to what extent do students’ mean MUSIC perceptions of a mathematics course relate to their mean instructor and course ratings? Recall that significant variability was attributed to students’ MUSIC perceptions across courses (see Table 5). We were able to explain a majority of this between-class variance, ranging from 61.8% for Usefulness to 95.8% for Interest (see Table 6), with class-level variables associated with course type, and mean expected grade, as well as mean instructor and course ratings. Here we discuss the modeling of this variance with these variables.

Class-level empowerment, interest, and caring were statistically and significantly related to mean *instructor* rating (whereas usefulness and success were not; see Figure 3 and Table 6). Class-level empowerment and interest were also statistically and significantly related to mean *course* rating (whereas usefulness and success were marginally significant [*p* < 0.10], and caring was not significantly related to course rating). These findings differ somewhat from the student-level relationships reported in a prior section in that usefulness and success were not significantly related to the instructor and course ratings at the class-level, but they were significantly related at the student-level. And, caring was not significantly related to course rating at the class-level, but it was at the student-level. To understand these statistical discrepancies, it is useful to examine Figure 3 more closely, starting with, for example, the relationship between caring and course rating. At the student-level, the relationship is statistically significant (β = 0.065, *p* < 0.01); yet, this value is not greater than the relationship at the class-level (β = 0.102, not significant). So, while some of the class-level variables were not found to significantly predict the class-level variance in MUSIC perceptions, our analysis of the compositional effects demonstrated that the magnitude of the student-level and class-level effects were not different, except for interest, which we explain in section “Compositional Effects.” Therefore, we conclude that the student-level and class-level effects are similar in direction (positively related) and magnitude (based on the analysis of the compositional effects), and are generally positive and statistically significant when controlling for expected grade, major (at the student-level), and type of course (at the class-level). In other words, in general, classes with higher instructor and course ratings tend to have more positive MUSIC perceptions. These findings indicate that students rate their instructor and course based, in part, on the motivational climate within the course (i.e., the aspects of the psychological environment that affect students’ motivation and engagement within a course, such as perceived empowerment/autonomy, usefulness/utility value, success expectancies, interest, and instructor caring).

Because students’ MUSIC perceptions are related to their instructional ratings, researchers should include these variables in other studies examining the effects of other variables on student ratings. For example, although students’ course grades are correlated with instructor ratings (Beran and Violato, 2005; Greenwald and Gillmore, 1997; Marsh and Roche, 2000), these relationships may be mediated or moderated by one or more of students’ MUSIC perceptions. Including all of these perceptions in one study provides a more multidimensional examination of how students’ perceptions affect their ratings.

### Compositional Effects

Our fourth research question asked: Are there compositional effects (i.e., differences between student- and class-level effects) associated with students’ instructor and course ratings? We only documented one compositional effect (*p* = 0.075) for these relationships: Students who were more interested in the course rated their instructor higher (note that the scale items referred to interest in the course and coursework, not interest in math more generally). However, we also found that the class-level effect was higher than the within-class effect. This means that students in courses with a higher mean instructor rating tend to rate their interest higher by virtue of being a member of that course (this is illustrated conceptually in Figure 2). The higher-rated instructors trigger more student interest in the course than the lower-rated instructors do. As an example specifically related to math courses, many liberal arts students may not have an individual interest in the discipline of mathematics, but could become interested in a mathematics course with a highly rated instructor. An implication is that instructor ratings could be used as a means to engage uninterested students in math courses by having more highly rated instructors teach math courses for non-math majors. This finding does not indicate that students will become more interested in the discipline of math, only that they would be more interested in and more likely to enjoy that particular math course.

### Limitations

One limitation of this study is that it was not designed to determine why students’ motivation-related course perceptions were associated with their instructor and course ratings. Another limitation is that we did not control for students’ beliefs that may have biased them in their instructor and course ratings. For example, it is possible that students’ ratings were biased based on the instructor’s gender, age, ethnicity, accent, or perceived attractiveness (Carpenter et al., 2020; Stroebe, 2020).

## Conclusion

Students’ class perceptions that are typically associated with student motivation (i.e., perceptions related to autonomy, utility value, expectancy for success, situational interest, and caring; Wentzel and Miele, 2016) are also related to their instructor and course ratings in undergraduate mathematics courses. These findings are consistent with current motivation theories (e.g., social learning theory, self-determination theory, expectancy-value theory, interest theories, and self-concept theories) in that students rate instructors and courses higher when they perceive that the course environment supports their autonomy, is useful for their goals, is one in which they can succeed, is interesting and enjoyable, and fosters caring relationships among students and the instructor. Therefore, we conclude that the motivational climate–created in part by the instructor–has an effect on students’ instructor and course ratings. Future research could examine the extent to which it is possible to affect the motivational climate and increase instructor and course ratings by using strategies that support students’ perceptions of autonomy/empowerment, usefulness, success, interest, and caring.

## Data Availability Statement

The raw data supporting the conclusions of this article will be made available by the authors, without undue reservation.

## Ethics Statement

The studies involving human participants were reviewed and approved by Virginia Tech Institutional Review Board. Written informed consent for participation was not required for this study in accordance with the national legislation and the institutional requirements.

## Author Contributions

All authors contributed to the conception and design of the study, cleaned and organized the database, contributed to manuscript revision, and approved the submitted version. LR collected data. JW performed the statistical analysis. BJ and JW wrote sections of the manuscript.

## Conflict of Interest

The authors declare that the research was conducted in the absence of any commercial or financial relationships that could be construed as a potential conflict of interest.

## Appendix A: Details of the Two-Level HLM

Here we present detailed descriptions of the student- and class-level equations. At the student level, we estimated a within-class (Level 1) equation (see Eq. 1) for each of the five perceptions of academic motivation. The Level 1 equation regressed perceptions of academic motivation on student-level variables. That is, academic motivation for student *i* in class *j* (MUSIC*_*ij*_*) was regressed on academic major (MAJOR), instructor rating (INSTRUCTOR), course rating (COURSE), and expected grade (GRADE).

(1) $${\text{MUSIC}}_{i\,j}=\beta_{0\,j}+\sum_{n=1}^{6}\beta_{n\,j}\,{\text{MAJOR}}_{n\,i\,j}+\beta_{7\,j}\,{\text{INSTRUCTOR}}_{i\,j}+\beta_{8\,j}\,{\text{COURSE}}_{i\,j}+\beta_{9\,j}\,{\text{GRADE}}_{i\,j}+r_{i\,j}$$

Each of the independent variables was group-mean centered (i.e., centered around the course mean), thus, β*_0j_* represents the unadjusted mean perception of academic motivation for class *j*. β*_*nj*_* is the differentiating effect associated with the six different major categories as compared to the mathematics major: Computer Science, Engineering/Physics, Life Science, Liberal Arts, Social Sciences, and Other; β*_7j_* is the effect of instructor ratings on student perceptions in class *j*; β*_8j_* is the effect of course ratings on student perceptions in class *j*; β*_9j_* is the effect of expected grade on student perceptions in class *j*; and *r*_*ij*_ represents the residual in the model and is assumed to be normally distributed with mean of 0 and a variance represented by σ^2^. Note that except for β*_0j_*, each of the effects β*_*nj*_* (for *n* = 1 to 9) represent the adjusted effect after controlling for the other student-level independent variables. In particular, GRADE was included to control for documented relationships between students’ expected grade and their course and instructor rating; similarly, MAJOR was included to control for potential differences in perceptions based on student focus of study.

At the class level, variability in mean academic motivation (β*_0j_*) was modeled by estimating a between-class (Level 2) equation (see Eqs 2–6) that regressed mean academic motivation on other class-level variables: course type (Advanced Mathematics, Non-STEM; Core STEM was the comparison variable), mean instructor rating for the class (MEAN INSTRUCTOR), mean course rating for the class (MEAN COURSE), and mean expected grade (MEAN GRADE). Note that INSTRUCTOR (β*_7j_*), COURSE (β*_8j_*), and GRADE (β*_9j_*) were initially allowed to randomly vary by class, and then later specified as non-randomly varying in the final model if not found to significantly vary by class. However, the variability in these predictors was not modeled.

(2) $$\beta_{0\,j}=\gamma_{00}+\gamma_{01}\,\left(\mathrm{Advanced}\,\mathrm{Math}\right)+\gamma_{02}\,\left(\text{Non-STEM}\right)+\gamma_{03}\,\left(\mathrm{MEAN}\,\mathrm{INSTRUCTOR}\right)\,+\gamma_{04}\,\left(\mathrm{MEAN}\,\mathrm{COURSE}\right)+\gamma_{04}\,\left(\mathrm{MEAN}\,\mathrm{GRADE}\right)+u_{0\,j},$$

(3) $$\beta_{n\,j}=\gamma_{n\,0}\;\left(\mathrm{for}n=1\mathrm{to}6\right),$$

(4) $$\beta_{7\,j}=\gamma_{70}+u_{7\,j},$$

(5) $$\beta_{8\,j}=\gamma_{80}+u_{8\,j},$$

(6) $$\beta_{9\,j}=\gamma_{90}+u_{9\,j}.$$

Each of the class-level predictors was grand mean centered. γ_00_ represents the average of the class means for each of the five perceptions of academic motivation across all classes; γ_*n0*_ (for *n* = 1 to 6) represents the average regression slope for each of the majors compared with mathematics majors for each of the five perceptions of academic motivation across all classes. Similarly, γ_70_, γ_80_, and γ_90_, represent the average slope between INSTRUCTOR, COURSE, and GRADE, respectively, and the perception of academic motivation across all classes. *u*_0j_, *u*_7j_, *u*_8j_, and *u*_9j_, represent the unique increment to the intercepts and respective slopes associated with class *j*.

## Appendix B: Further Description of Compositional Effects

Here we provide further conceptual discussion of compositional effects presented in Figure 2. In Figure 2, we represent three hypothetical classes that differ by one unit in mean instructor rating (e.g., the mean instructor ratings for classrooms A, B, and C are 3, 4, and 5, respectively). Note, at the same time, the expected within-class increase in interest (represented on the *y*-axis), shown with a dotted line, for two students in classroom A differing by one unit on their instructor rating (again 3 and 4). Now, think about two students each with an instructor rating of 4, but one is in class A and the other in class B (differing by one unit in mean instructor rating). The expected increase in interest for the student in class B over the student in class A represents the compositional effect, resulting from membership in class B instead of class A. In comparison to the situation represented in Figure 2, it is also possible that the student- and class-level effects are both negative and that the compositional effect represents an additional decrease or increase in effect associated with the course. It is also possible that the compositional effect represents a change in direction of the effect; in other words, the within-class effect is positive and the between-class effect is negative (or vice versa).
